# Noncoding RNAs as Novel Biomarkers in Prostate Cancer

**DOI:** 10.1155/2014/591703

**Published:** 2014-08-28

**Authors:** C. G. H. Rönnau, G. W. Verhaegh, M. V. Luna-Velez, J. A. Schalken

**Affiliations:** ^1^Department of Urology, Radboud University Medical Center, Post 267, P.O. Box 9101, 6500 HB Nijmegen, The Netherlands; ^2^Department of Urology, Universitätsmedizin Greifswald, Ferdinand-Sauerbruch-Strasse, 17475 Greifswald, Germany; ^3^Radboud Institute for Molecular Life Sciences, P.O. Box 9101, 6500 HB Nijmegen, The Netherlands

## Abstract

Prostate cancer (PCa) is the second most common diagnosed malignant disease in men worldwide. Although serum PSA test dramatically improved the early diagnosis of PCa, it also led to an overdiagnosis and as a consequence to an overtreatment of patients with an indolent disease. New biomarkers for diagnosis, prediction, and monitoring of the disease are needed. These biomarkers would enable the selection of patients with aggressive or progressive disease and, hence, would contribute to the implementation of individualized therapy of the cancer patient. Since the FDA approval of the long noncoding *PCA3* RNA-based urine test for the diagnosis of PCa patients, many new noncoding RNAs (ncRNAs) associated with PCa have been discovered. According to their size and function, ncRNAs can be divided into small and long ncRNAs. NcRNAs are expressed in (tumor) tissue, but many are also found in circulating tumor cells and in all body fluids as protein-bound or incorporated in extracellular vesicles. In these protected forms they are stable and so they can be easily analyzed, even in archival specimens. In this review, the authors will focus on ncRNAs as novel biomarker candidates for PCa diagnosis, prediction, prognosis, and monitoring of therapeutic response and discuss their potential for an implementation into clinical practice.

## 1. Introduction

### 1.1. Prostate Cancer Diagnosis

The prostate is an exocrine gland in the male reproductive system that is responsible for the production of 50–70% of the seminal fluids. In men, prostate cancer (PCa) is the second most common diagnosed malignant disease and the sixth leading cause for cancer related death among men worldwide, with an estimation of 899.000 new cases and 258.000 deaths in 2008 [1, 2]. The rate of PCa diagnosis increased over the past decades due to an aging population, increased awareness, and the use of prostate-specific antigen (PSA) in serum for screening and diagnosis [3]. Upon abnormal digital rectal examination (DRE) and/or elevated serum PSA values, the diagnosis of PCa is usually obtained by pathological evaluation of transrectal ultrasound-guided prostate needle biopsies. However, this procedure is limited by false-negative biopsies and overdiagnosis of clinically insignificant malignancies [4, 5]. PCa is a heterogeneous disease and the clinical behavior ranges from slow-growing tumors with no or little clinical significance to aggressive metastatic and lethal diseases. By definition, clinically insignificant PCa does not contribute to PCa mortality [6] and the treatment of indolent PCa can result in side effects that reduce quality of life of the patient for no or little benefit.

The use of biomarkers has the potential to improve the diagnosis of cancer, especially to identify cancer at an early stage of disease with potentially curative therapy options. The currently used PSA test has some well-known limitations [7]. Although PSA is prostate-specific, it is not cancer-specific due to elevated levels of serum PSA under benign conditions, like benign prostate hyperplasia, urinary retention, prostatitis, trauma, or physical manipulation [7]. Approximately, 30% of men with a serum PSA of 5–10 ng/mL and >50% of men with a PSA >10 ng/mL will have prostate cancer. More importantly, clinical trials have shown that the PSA testing and screening is associated with an overdiagnosis and as a consequence an overtreatment of patients with indolent disease [4, 5, 8].

One of the current clinical priorities includes the identification of biomarkers that discriminate between indolent and aggressive PCa so that patients with an indolent disease with low risk of progression may better benefit from avoiding unnecessary treatments. The aim should as well be to identify patients with an aggressive, rapidly lethal PCa at an early stage, for which potential curative therapy options are available. Without any doubt, there is a need for new diagnostic and predictive biomarkers, and these markers would enable individualized therapeutic management for the cancer patient.

### 1.2. Prostate Cancer Monitoring

Beside screening and diagnosis, serum PSA is currently also used for monitoring disease progression. PCa depends on androgen receptor activity at all stages. Standard therapy of disseminated prostate cancer in hormone-naive patients is based on androgen-deprivation therapy or androgen receptor antagonists. Unfortunately, successful treatment effects are often followed by recurrence of PCa, resulting in the so-called castration-resistant prostate cancer (CRPC). Currently, there are no curative treatments for CRPC available. Docetaxel is the first-line chemotherapy for CRPC, providing modest survival benefits [9–11]. Response to treatment is usually determined by changes in serum PSA levels and reduction of tumor burden on radiological scans. The response rate is ~50%, and many patients suffer from significant toxicity [9, 11]. Metastasis and chemoresistance are reasons for the mortality of PCa patients. There are advances in the development of alternative effective therapies (e.g., abiraterone acetate, enzalutamide, cabazitaxel, and radium-223), but there is still a lack of useful biomarkers for the management or monitoring of patients with CRPC. Absolute serum PSA values are not suitable for and predictive of CRPC patients. Predictive biomarkers would be used to identify patients who most likely benefit from a particular treatment, enabling personalized medicine and hence treatment failure including side effects could be avoided.

### 1.3. Biomarkers

The heterogeneous nature of PCa and CRPC is coupled with genetic and epigenetic alterations that occur during disease progression and response to therapy. These changes can lead to the expression or production of novel disease-specific macromolecules, which could serve as novel biomarkers. Biomarkers are molecules that can provide information about the disease. In general, beside their role in diagnosis, they could be useful for the evaluation of the disease predisposition, screening, prognosis, prediction of drug response, monitoring, and pharmacodynamic properties (e.g., for a determination of the most effective dose) in combination with clinical history and parameters [12]. Biomarkers should be detectable in tissue, obtained by biopsy or surgical resection, or in bodily fluids like blood, urine, and semen. Potential biomarkers can be specific cells, proteins (enzymes, hormones, etc.), metabolites, DNA or an epigenetic modification of DNA, and expression levels of (novel) RNA transcripts, including noncoding RNAs (ncRNAs) [13].

Human prostatic acid phosphatase (PAP) was the first reported biomarker for PCa [14]. Proteomics and genomic technologies have enhanced the discovery of potential novel candidates. Many excellent reviews have summarized novel candidate biomarkers including proteins (e.g., α-methylacyl-CoA racemase, endoglin, prostate-specific membrane antigen [PSMA], caveolin-1, interleukin-6, CD147, TGF-*β* 1, and human kallikrein-2), genetic biomarkers (e.g.,* TMPRSS2:ETS* gene fusions,* BRCA1/2* mutations), epigenetic modification (e.g., methylation of the glutathione S-transferase [*GSTP1*] gene and histone modifications), and expression of (novel) mRNA transcripts in PCa [6, 15–19]. Examples are shown in Table 1. Despite technological advances that contributed to the identification of novel biomarker candidates in serum, the use of proteins as biomarkers is still limited by the wide range of protein concentrations. In addition, it is often difficult to detect low-abundant proteins, due to interfering compounds, the masking effects of high-abundance proteins, high levels of salts, and big variations between individuals [6]. In contrast, for example, RNA-transcripts can be reproducibly detected and quantified in all kinds of specimens, even when present at very low levels due to possibility to include amplification steps.

Common genetic alterations in tumors also have an impact on specific noncoding RNAs (ncRNAs). NcRNAs (e.g., microRNAs) have received increasing attention of investigators as they can target multiple signaling pathways related to tumor progression, invasion, metastasis, and chemoresistance. Recent evidence suggests that ncRNAs represent useful and promising markers for diagnostic and prognostic purposes alone or in addition to other candidate markers. NcRNAs have the potential to improve the current tests or even to be superior to established protein-based biomarkers. Therefore, the authors of this review focus on ncRNAs and discuss their potential as new biomarkers for PCa diagnosis, prognosis, prediction, and monitoring of PCa patients.

### 1.4. Noncoding RNAs

**Figure 1:**
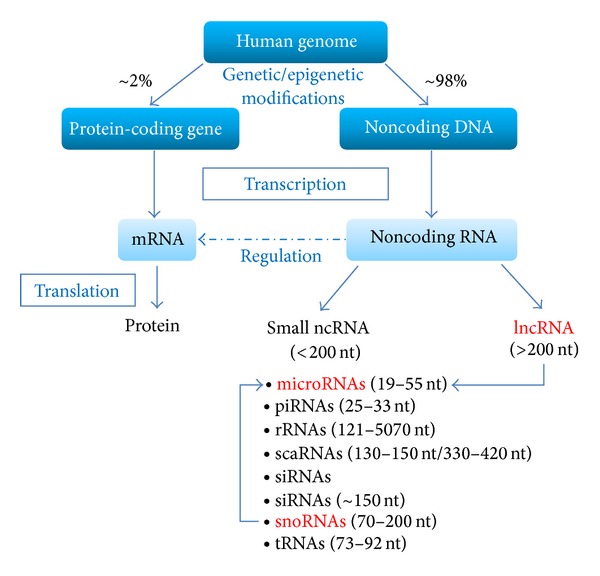
The human genome consists of approximately 2% protein-coding sequences, which can be transcribed into messenger RNAs (mRNAs) and then translated into proteins. The majority of the human genome exists in nonprotein-coding DNA, which can be transcribed in (functional) noncoding RNAs (ncRNAs). According to their size and function, ncRNAs can be grouped into long noncoding RNAs (lncRNAs) and small ncRNAs. The group of small ncRNAs, which are less than 200 nucleotides (nt) in length, consists of microRNAs (miRNAs), piwi-interacting RNAs (piRNAs), ribosomal RNAs (rRNAs), small Cajal body-specific RNAs (scaRNAs), small-interfering RNAs (siRNAs), small nuclear RNAs (snRNAs), small nucleolar RNAs (snoRNAs), and transfer RNAs (tRNAs). Beside their biogenesis from hairpin precursor molecules, miRNAs can also be derived from lncRNAs and snoRNAs (highlighted in red).

Only about 25,000 protein-coding genes (covering approximately 2% of the human genome) have been recognized by the International Human Genome Sequencing Consortium [20]. Most of the human genome does not code for any protein and therefore is called noncoding DNA (ncDNA). Most of the ncDNA, estimations ranging from 60 to 90%, is transcribed into functional ncRNAs. These ncRNAs are not translated into proteins [21, 22] but still act as important mediators of gene regulation in physiological as well as pathological processes. Cumulative evidence points towards an important role of ncRNAs in cancer initiation, development, and progression [22]. NcRNAs are classified in small and long ncRNAs (lncRNA) based on their size and function (Figure 1).

#### 1.4.1. Long Noncoding RNAs

Long noncoding RNAs (lncRNAs) have a length greater than 200 nucleotides (nt) and are located in the nucleus or in the cytoplasm. The number of lncRNAs is not clear. It has been estimated that approximately 15,000 lncRNAs are present in the human genome, but the GENCODE v19 catalog of human lncRNAs contains 13,870 lncRNA genes that produce 23,898 lncRNAs [23]. However, only a few lncRNAs are expressed in a cell type-specific manner [24, 25]. Recent studies have demonstrated that lncRNAs regulate many processes such as transcription, translation, cellular differentiation, gene expression regulation, cell cycle regulation, chromatin modification, and nuclear-cytoplasmic trafficking [20, 26–28]. LncRNAs can function as oncogenes or as tumor suppressors. Examples for oncogenic lncRNAs are* CDKN2B-AS1 *(ANRIL), which is described in tissue of melanoma and PCa, HOX antisense intergenic RNA (*HOTAIR*), which was found in breast and colon cancer, and* CCAT1*, which is increased in gastric carcinoma tissue. LncRNAs that function as tumor suppressors are, for example, growth arrest-specific 5 (*GAS5*), which is aberrantly expressed in several cancers including PCa and the pseudogene* PTENP1* [20]. Several PCa-specific lncRNAs have been identified, such as* PCA3*,* PCAT1*, and* PCGEM1.* The identification and function of these PCa-specific lncRNAs are excellently reviewed by Walsh et al. [23]. The most interesting lncRNA candidates as PCa biomarkers are discussed in the chapter “lncRNAs and prostate carcinogenesis” (see Section 2.1).

#### 1.4.2. Small Noncoding RNAs

Small ncRNAs, in general, have a size of less than 200 nucleotides. Based on their size and function the small ncRNAs can be subdivided into microRNAs (miR, miRNAs), piwi-interacting RNAs (piRNAs), ribosomal RNAs (rRNAs), short interfering RNAs (siRNAs), small Cajal body-specific RNAs (scaRNAs), small nuclear RNAs (snRNAs), small nucleolar RNAs (snoRNAs), and transfer RNAs (tRNAs, Figure 1).

MiRNAs are the best characterized class of small ncRNA transcripts. Currently, there are 2,578 mature human miRNAs listed in the miRBase catalog of human miRNAs (v20, June 2013) [22, 29], and the number of identified miRNAs is still rising. Many excellent articles have described the biogenesis and function of miRNAs and their role in human diseases such as cancer. MiRNAs are fragments of single-stranded ncRNAs of 19–25 nt, derived from hairpin precursor molecules [30, 31]. They are reported to regulate more than 50% of all protein-coding genes in mammalian cells [31, 32]. Predominantly, they repress protein expression by inhibiting translation or by degradation of the target mRNA [33]. Genes can be targeted by multiple miRNAs and each miRNA is able to target hundreds of mRNAs directly or indirectly. According to their important role in the regulation of genes that are involved in many physiological processes, it is not of a surprise that they are also involved in the initiation and progression of cancer [34]. The dysregulation of miRNAs has been demonstrated in all types of human malignancies. Like, for proteins, both miRNAs with oncogenic functions, also called oncomirs, and tumor suppressive miRNAs have been found [34, 35]. The expression of miRNAs can be influenced by chromosomal rearrangements (deletions, amplifications, and mutations), by DNA methylation, or by other types of transcriptional control.

It is becoming evident that also another class of small ncRNAs, the small nucleolar RNAs or snoRNAs, may be dysregulated in cancer. Most snoRNAs are ubiquitously expressed and function in the maturation and modification of other ncRNAs such as rRNAs [36]. SnoRNAs can be divided into two structural classes, C/D-box (SNORD) and H/ACA-box (SNORA) RNAs [37]. Until now, approximately 400 snoRNA species have been identified in the human genome [37]. It is also known that some miRNAs originate from snoRNAs and these snoRNA-derived miRNAs are termed sno-miRs, sno-miRNAs, or sdRNAs. Very recently it was reported that snoRNAs and fibrillarin, which is an enzymatic small nucleolar ribonucleoprotein (snoRNP), are frequently overexpressed in human breast and prostate cancer tissues [38]. Specific cancer-related snoRNA signatures in blood were described for nonsmall-cell-lung cancer [37, 39]. Further investigations are necessary to confirm these findings, to describe the role of snoRNAs and sdRNAs in (prostate) cancer, and to evaluate their potential as biomarker.

## 2. What Is Known about Noncoding RNAs in Prostate Cancer?

### 2.1. Long Noncoding RNAs and Prostate Carcinogenesis

A well investigated lncRNA in PCa is* PCA3*.* PCA3* was found to be strongly overexpressed (66-fold in PCa tissue compared to normal prostate tissue) in more than 95% of primary PCa specimens and metastasis [40, 41]. It is not expressed in other normal human tissues and, therefore,* PCA3* is so far the most PCa-specific gene known.* PCA3* can be identified in post-DRE voided urine, and based on this it was claimed to be a novel PCa biomarker. The PROGENSA PCA3 test is the first FDA-approved urine-based molecular diagnostic test for men with elevated serum PSA and a previous negative biopsy. A urine PCA3 score (*PCA3*-to-*PSA* ratio) with a cut-off of ≥35 had an average sensitivity of 66% and a specificity of 76% for the prediction of PCa at biopsy (area under the curve (AUC) 0.75), while serum PSA had only a specificity of 47% [18, 42]. The PCA3 score is not influenced by age, inflammation, prostate volume, or 5α-reductase inhibitors [18]. The use of the* PCA3* ncRNA as biomarker in clinical practice has been extensively reviewed [18, 43]. However, in independent studies, no significant association of PCA3 score in urine with prognostic or predictive parameters was found.

Additional PCa-specific lncRNAs have been described (Table 1). Overexpression of oncogenic lncRNAs may promote tumor-cell proliferation and metastasis, and aberrant expression of lncRNAs in PCa is associated with disease progression. The lncRNA metastasis-associated lung adenocarcinoma transcript 1 (*MALAT1*) is overexpressed in several human cancers, including PCa. In primary PCa,* MALAT1 *overexpression is correlated with markers of poor prognosis (high Gleason-score, higher tumor-node-metastasis (TNM) stage, and serum PSA >20 ng/mL) and its expression significantly increases from hormone sensitive PCa to CRPC [44]. Small interfering RNA- (siRNA-) mediated knockdown of* MALAT1* in PCa cell lines 22Rv1 and LNCaP inhibits cell growth, invasion, and migration and results in cell cycle arrest in the G0/G1 phase, demonstrating its functional role in PCa [44].

The lncRNA prostate cancer associated ncRNA transcript- (*PCAT-*) 1 is highly prostate-specific and is upregulated in a subset of high-grade localized (Gleason-score ≥7) and metastatic PCa [23].* PCAT1 *induces cell proliferation and has a repressive effect on gene expression, for example, on the tumor suppressor* BRCA2 *[45]. By Affymetrix array-based expression profile analysis 213 lncRNAs were found to be significantly differentially expressed in PCa tissue compared to benign prostate tissue. Based on these results, two novel clinically relevant lncRNAs have been identified in PCa,* PCAT6*, and* PCAT7. *The expression of both lncRNAs increased from normal prostate tissue to primary to metastatic PCa [23]. Knockdown of* PCAT6 *and* PCAT7 *reduces cell growth and soft agar colony formation in LNCaP cells.

Second chromosome locus associated with prostate-1 (*SCHLAP1*) is a lncRNA that is highly expressed in 25% of prostate tumors.* SCHLAP1* expression is revealed as a predictor for PCa aggressiveness, with highly significant hazard ratios for predicting biochemical recurrence, clinical progression, and PCa-specific mortality in a large cohort of localized PCa (*n* = 235, median follow-up 8.1 years).* SCHLAP1 *has the potential as prognostic marker due to a significant association between biochemical recurrence and overall survival and high expression levels of* SCHLAP1 *[23, 46].

The lncRNA prostate cancer gene expression marker 1 (*PCGEM1*) was first described by Srikantan et al. in 2000 [47]. Recently, Yang et al. reported about two lncRNAs, prostate cancer noncoding RNA1 (*PRNCR1*) and* PCGEM1*.* PCGEM1* is overexpressed in more than half of PCa tissues [48]. The authors reported that these lncRNAs regulate AR-mediated gene transcription in PCa. In CRPC, both lncRNAs* PCGEM1* and* PRNCR1 *activate the transcription of AR splicing variants, even in the absence of ligand binding [23, 48]. However, Prensner et al. confirmed the association of* PCGEM1* and* PRNCR1* with PCa, but the authors found no interaction with AR or components of AR signaling. Furthermore, they demonstrated that* PCGEM1* and* PRNCR1* are not useful as prognostic marker after analysis of 230 high-grade PCa patients and their clinical outcome [49]. It is of note that it is also recently reported that* PCAT18 *is a highly prostate-specific transcript, upregulated in prostate cancer, and that the expression of* PCAT18 *is induced by AR signaling [50]. Furthermore the lncRNA CBR3 antisense RNA 1 (*CBR3-AS1*) has been reported to be associated with changes in AR activity [51].

So, in addition to* PCA3*, several novel PCa-specific or PCa-associated lncRNAs are on the horizon, but none of these, so far, made it to a clinical test for PCa. In initial studies, the presence of lncRNA fragments in blood specimens has been analyzed. For example,* MALAT1 *levels in plasma were determined and these were found to be able to distinguish biopsy-positive from biopsy-negative PCa patients (AUC 0.767; *P* < 0.001). However, the sensitivityof the plasma* MALAT1 *test was 58.6% and therefore lower than that for serum PSA [44]. Research concerning lncRNAs as biomarkers for PCa is still in its infancy, and further investigations as well as large validation studies are necessary before a translation into the clinical setting will be possible.

### 2.2. Dysregulated microRNAs in Cancer Tissue

Many miRNA profiling studies in PCa have been performed, using technologies such as microarray analysis and next-generation RNA sequencing (NGS) [52]. These methods allow the analysis of many miRNAs simultaneously, but because of their low sensitivity and their high throughput-screening nature a validation in independent samples using quantitative technologies such as qRT-PCR is needed.

Some miRNAs (Table 1) have been shown to be dysregulated and functionally relevant in certain cancer types [53, 54]. Volinia et al. reported about 21 miRNAs that were upregulated in six solid cancer tissues [55]. MiR-21 is well known to play an important role in normal and pathological processes including development, inflammation, cardiovascular function, and cancer. Many researchers found high expression of miR-21 in almost all types of solid cancer tissues including PCa and, therefore, it was classified as an oncomir [56]. MiR-21 targets tumor suppressor genes, such as phosphatase and tensin homolog (*PTEN),* tumor suppressor gene tropomyosin 1 (*TPM1*), programmed cell death 4 (*PDCD4*), maspin, and reversion-inducing cysteine-rich protein with Kazal motifs (*RECK*) [57–60]. The activation of miR-21 might enhance general processes of tumor progression, invasion, and metastasis [17]. Furthermore, Wang and Zhang reported that miR-21 was elevated in serum of patients with breast cancer, colorectal cancer, lung cancer, esophageal cancer, and gastric cancer compared to healthy controls and concluded that miR-21 has the potential as a broad-spectrum serum-based biomarker for the detection of some solid cancers. However, the authors found no correlation with gender, clinical stage, and lymph node metastasis [61].

Zhang et al. [54] systematically reviewed 49 studies that investigated the expression of miR-183 family members, consisting of miR-96, miR-182, and miR-183 in different tumors (HCC, gastric, pancreatic, colon, rectal, breast, gynecologic, lung, bladder, and prostate cancer) compared to noncancerous tissues. Several studies reported that these miRNAs are directly involved in human cancer processes, such as cellular differentiation, tumorigenesis, proliferation, apoptosis, and metabolism [54, 62–64]. All miRNAs from the miR-182-96-183 cluster, located on chromosome 7q32.2, were upregulated in 14 human cancers. The miR-183 family members were most upregulated in colorectal and prostate cancer tissue [65–68], followed by bladder, lung cancer, and HCC tissue [54]. The results for breast and gastric cancer were inconsistent and miR-183 was downregulated in osteosarcoma [69]. These miRNAs were so far not described in the circulation of cancer patients, but higher levels of miR-96, miR-182, and miR-183 were found in urine specimens of bladder cancer patients compared to healthy controls [70, 71].

Upregulation of miR-221 and miR-222 has been observed in several cancer cells [72] and it is also reported that the miR-221/miR-222 cluster is highly expressed in metastatic CRPC tissue. A transient overexpression of miR-221/miR-222 in LNCaP cells promoted the development of a CRPC-like phenotype. As a result of elevated expression of miR-221, expression of many cell cycle genes was altered and pathways promoting epithelial-to-mesenchymal transition (EMT) and tumor metastasis were activated [72]. Upregulated levels of circulating miR-221 were identified in serum of PCa patients and also in serum or plasma of patients, for example, with lung cancer, ovarian cancer, melanoma, and lymphoma, compared to their healthy controls [31, 73].

MiR-205 is frequently downregulated in different cancers, including glioblastoma [74, 75], melanoma [76], breast cancer [77, 78], renal cell carcinoma [79], and prostate cancer [80–83]. The presence of miR-205 suppresses cell proliferation and metastasis. In prostate cancer, miR-205 functions as a tumor suppressor through downregulation of multiple targets like BCL2 [81], protein kinase C epsilon [84], and androgen receptor [85]. Genes regulated by miR-205 are enriched in, for example, the MAPK/ERK, toll-like receptor, and IL-6 signaling pathways [85]. Furthermore, miR-205 is observed to be downregulated in cells that have undergone EMT, a process that is accompanied by a decrease in E-cadherin and fibronectin expression [86]. Inversely, expression of miR-205 is upregulated in mesenchymal cells that initiated mesenchymal-to-epithelial transition (MET) associated with an upregulation of E-cadherin and a reduction of tumor cell migration and cell invasion [82, 85]. MiR-205 inhibits tumor invasion through several pathways; for example, low-density lipoprotein receptor-related protein 1 (LRP-1) promotes cancer cell migration and invasion by inducing the expression of matrix metalloproteinases (MMP) 2 and 9 [87, 88]. Schaefer et al. reported about an inverse correlation between prostate tissue miR-205 levels and the occurrence of metastases and shortened overall survival of PCa patients [66, 85]. Wang et al. confirmed these results and they reported a stronger downregulation of miR-205 in advanced and/or metastatic PCa [89]. MiR-205 was significantly downregulated in serum of breast cancer patients compared to healthy controls [90], but studies on serum of PCa patients are lacking. Nevertheless, circulating miR-205 may be a promising biomarker for PCa prognosis.

In addition to miR-205, miRNAs from the miR-143/miR-145 cluster are also downregulated in (prostate) cancer [91]. MiR-143 and miR-145 are transcribed after TGF*β*1 pathway activation and inhibition of this pathway will lead to a decrease of these miRNAs [92, 93]. MiR-143 targets Kirsten rat sarcoma viral oncogene homolog (KRAS), ELK1, myosin 6, B cell lymphoma 2 (BCL-2), and extracellular signal-regulated kinase 5 (ERK). A loss of miR-143 expression causes an upregulation of ERK5, which induces cell proliferation, survival, and invasion and as a consequence it leads to the development of more aggressive forms of PCa [92]. The downregulation of miR-145 also leads to enhanced cell proliferation. MiR-145 targets MYO6 and fascin homolog 1 (FSCN1), proteins also associated with PCa progression [92]. Whether miR-143 and miR-145 can serve as biomarker for PCa still has to be investigated.

While the above described miRNAs are dysregulated in many types of cancer, some miRNAs seem to be tissue-specific. MiRNA expression profiles specific for PCa have been discovered [17, 55, 66, 91, 94–98]. These profiling studies showed differences in the expression of miRNAs in localized or metastatic prostate cancer compared to benign prostate epithelium or BPH, but in these studies also a large number of non-tissue-specific miRNAs were found. The first profiling of miRNAs in PCa was published in 2007. Porkka et al. [94] observed a downregulation of 37 miRNAs and an upregulation for 14 miRNAs in PCa. Schaefer et al. identified 10 miRNAs (miR-16, miR-31, miR-125b, miR-145, miR-149, miR-181b, miR-184, miR-205, miR-221, and miR-222) to be downregulated and 5 miRNAs (miR-96, miR-182∗, miR-183, and miR-375) that were upregulated in PCa tissues compared to the matched normal tissues [66]. Ozen et al. found a widespread downregulation of miRNAs in prostate cancer tissue [96] and Ambs et al. reported about miR-32 to be upregulated in PCa tissue [95]. Many of these differentially expressed miRNAs are described only in one study and, thus, have to be validated in independent investigations.

### 2.3. Circulating microRNAs as Diagnostic and Prognostic Biomarkers in Prostate Cancer

For most of the deregulated miRNAs in tissue, it is still unclear whether they can serve as novel diagnostic and/or prognostic biomarkers. However, some of the dysregulated ncRNAs in cancer tissue can be analyzed in body fluids and are therefore promising as putative biomarkers for PCa (Table 1).

**Figure 2:**
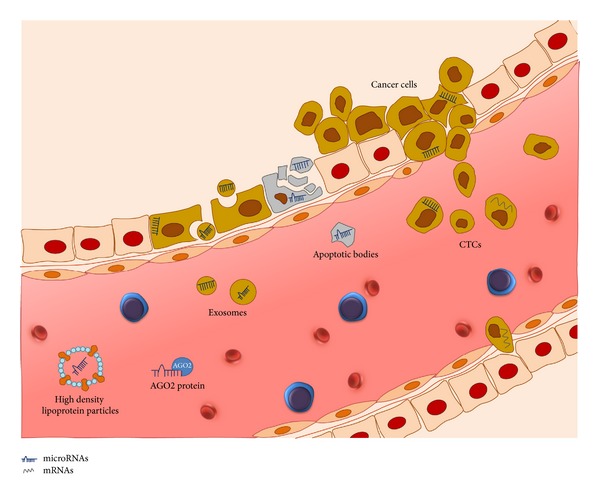
microRNAs can be released from normal and malignant cells in the blood circulation. This process can be passive, for example, after apoptosis, or as active secretion. In plasma or serum, cell-free microRNAs are protected against RNase activity, for example, through their binding to AGO1, AGO2 proteins or to high density lipoproteins. Furthermore, microRNAs can also be incorporated into microvesicles, such as exosomes, apoptotic bodies, or circulating tumor cells (CTCs). Specific microRNAs released from prostate cancer cells in body fluids, such as blood or urine, can serve as novel biomarkers for diagnosis, prognosis, prediction, or monitoring of cancer patients.

MiRNAs can be released into the blood circulation (Figure 2) as a result of apoptotic and necrotic cell death as well as by active secretion [99]. Extracellular miRNAs may be involved in cell-cell communication and immune regulation [100]. Cell-free miRNAs are detectable in all body fluids. This accessibility makes them attractive as promising biomarker candidates for PCa, as well as for other malignancies. Mitchell et al. showed that cell-free circulating miRNAs are highly stable and reproducibly detectable in serum and plasma of humans [101, 102]. The stability of miRNAs in noncell environments (i.e., protection from RNase activity) may be because they form complexes with RNA-binding proteins such as AGO1, AGO2 or high-density lipoprotein (HDL) for the transport of miRNAs to recipient cells via the bloodstream (Figure 2). Beside other protective mechanism, they are also incorporated in microvesicles, like exosomes and apoptotic bodies [103–105]. Circulating miRNAs also may originate from the tumor surrounding tissue, from other tissues in the human body that are not involved in cancer or from blood cells [106, 107]. Studies are ongoing to characterize potential cancer-specific circulating miRNAs.

Some studies described an upregulation of miR-21, miR-200c, and miR-375 in prostate cancer tissue compared to nonmalignant prostate tissue [66, 95, 108, 109]. Higher levels of miR-21, miR-141, miR-200a, miR-200b, miR-200c, and/or miR-375 were also observed in serum, plasma, or circulating microvesicles in patients with metastatic disease compared to localized PCa or healthy controls [109–113]. The latter findings suggest that these miRNAs are derived from tumor tissue and may act as circulating miRNA biomarkers for the detection of metastatic disease. Circulating miRNAs can originate from tumor cells, from other affected organs involved in tumor invasion and metastasis, or from inflammatory responses [114]. MiRNAs have been associated with prostate cancer; for example, miR-21 and miR-221 and their deregulation were described in tumor tissue [55, 66, 94, 95, 98] and serum of PCa patients [110, 115, 116]. The differences in serum miRNA levels between PCa patients (*n* = 51) and healthy controls (*n* = 20) were highly significant for miR-21 (*P* < 0.001, AUC 88%) and for miR-221 (*P* < 0.001, AUC 83%). In patients with metastatic disease the serum levels of miR-21, miR-221, and miR-141 (*P* < 0.001, AUC 75.5%) were significantly higher than in patients with a localized PCa [115]. Furthermore, circulating miR-21 and miR-221 were reported to discriminate PCa patients with intermediate risk from those with low risk CAPRA scores with a sensitivity of 38.1% and a specificity of 94.2% (AUC 0.801) [116].

Across independent studies, circulating miR-141 and miR-375 were the most promising miRNAs, suggested as diagnostic and prognostic markers for high risk PCa, and two miRNAs that were also decribed to be higher expressed in prostate cancer tissue compared to nonmalignant prostate tissue [66, 91, 95, 113]. Four independent studies analyzed circulating miRNA levels in about 240 PCa patients and 70 healthy controls in total and found either miR-141 or miR-375 or both as a diagnostic and prognostic marker(s) [17, 101, 113, 115, 117]. Mitchell et al. [101] reported, as the first, that tumor-derived miRNAs can enter into the circulation and can be measured in serum and plasma as a blood-based biomarker for human cancer. They observed that circulating miR-141 was significantly elevated in sera of prostate cancer patients (sensitivity 60%, specificity 100%) [101]. Brase et al. confirmed that circulating miR-141 and miR-375 were highly detectable in serum from patients with an advanced disease, and their levels correlated with high-risk tumors (Gleason-score ≥8 or metastasis) [113].

Independent studies evaluated the increased serum levels of miR-141 in patients with aggressive PCa [113, 115, 118, 119], indicating its diagnostic potential. However, Westermann et al. recently analyzed serum miR-141 in a multicenter study and reported that miR-141 did not qualify as an early diagnostic marker for PCa (AUC 0.49). In this study, serum samples were collected from 170 patients who underwent a prostate biopsy, of which 54 patients were diagnosed with PCa. MiR-141 levels were not increased in serum from patients with evident PCa compared to patients with a PCa-negative biopsy, but the authors reported that miR-141 levels were significantly increased in patients with a higher Gleason-score (*P* = 0.049). However, there was no association with clinical tumor stage or PSA [120]. The function of miR-141 and miR-375 in prostate cancer is still unclear. Waltering et al. found that miR-141 was upregulated by androgens and that an overexpression of miR-141 in LNCaP cells increased cell proliferation, suggesting that miR-141 could be involved in PCa progression [121]. Recently, it was reported that upon androgen treatment increased levels of miR-141 and miR-375 were released in the cell culture medium of LNCaP cells [122]. The authors concluded that an increased release of miR-141 and miR-375 from androgen-stimulated cells may explain their higher levels in the blood of patients with advanced PCa or CRPC [122, 123].

Beside single miRNAs, Chen et al. reported about a panel of five circulating miRNAs (miR-30c, miR-622, miR-1285, miR-let7c, and miR-let-7e) to discriminate PCa from healthy individuals and BPH patients with a sensitivity of 61% to 90% and a specificity of 57% to 75%, respectively (PCa versus healthy control AUC 0.86 and PCa versus BPH AUC 0.924) [124].

Larne et al. [68] identified four miRNAs that discriminate PCa from nonmalignant tissue. They combined these four miRNA levels into a miRNA index quote (miQ): (miR-96-5p × miR-183-5p)/(miR-145-5p × miR-221-5p). The advantages of such a miQ are the increased discriminatory power of the test and omitting the need for housekeeping genes. The described miQ test predicts the presence of PCa (*P* < 0.0001) with high accuracy (AUC 0.931), which was verified in four independent cohorts. miQ has also a prognostic power to predict aggressiveness of tumors (AUC 0.895), metastatic status (AUC 0.827), and overall survival (*P* = 0.0013, hazard ratio 6.5) [68]. In this pilot project, miQ was used with miRNAs identified in tissue specimens, but it has also the potential for analysis of a panel of miRNAs in serum/plasma and urine. The value of miQ as a PCa biomarker has to be validated in independent multicenter validation studies.

In recent investigations, additional up- or downregulated miRNAs in serum or plasma were described. Most of these miRNAs were only reported in one study, and therefore large validation studies are needed to verify these putative candidates [112, 116, 124–130].

In summary, miR-141 and miR-375 are the most consistently reported circulating miRNA candidates to be associated with high risk PCa. However, inconsistent findings based on miRNA levels are also notable. Major reasons for the variations and the lack of consistency in the data of circulating miRNAs are potentially variability in methodology of extraction and quantification of (micro) RNAs and data analysis [131]. There is no consensus on suitable reference RNAs that could be used as internal controls. Current protocols, using spiked-in synthetic nonhuman (e.g.,* Caenorhabditis elegans*) miRNAs only correct for technical variability [101, 132]. Furthermore, contamination of miRNAs due to haemolysis or during sample processing is possible. The studies, published until now, are a solid basis for discovery and establishing new biomarkers in PCa, but for further studies better, widely accepted, standardization, and normalization protocols are needed.

### 2.4. Urinary microRNAs as Diagnostic and Prognostic Biomarkers

Due to its noninvasive and easily attainable nature, urine is a promising substrate for biomarker testing. It is known that prostate cells and ncRNAs can be directly released into the urethra through the prostatic ducts after DRE. Cellular and extracellular miRNAs in urine and urinary microvesicles may be derived from urological cancers (e.g., prostate, bladder, or renal cancer), but they can originate from normal epithelium, the glomerulus, or renal tubules [133]. Therefore, specific markers and good normalization procedures are needed. The PROGENSA urine test for PCa makes use of such a PCa-specific biomarker, the* PCA3* lncRNA, and a normalization procedure using the prostate epithelium-specific marker* KLK3* (see Section 2.1).

Until now, only 4 studies reported miRNA levels in urine of PCa patients [112, 134–136]. Bryant et al. found that miR-107 and miR-574-3p were present at significantly higher levels in urine of men with PCa compared to controls [112]. Srivastava et al. analyzed miR-205 and miR-214 in tissue and urine of PCa patients and reported that these two candidates were present in detectable levels in urine samples but significantly lower in the cancer group compared to healthy controls. In this study, urinary miR-205 and miR-214 were able to discriminate PCa patients and healthy controls with 89% sensitivity and 80% specificity [134]. In a profiling study using microarray analysis and validation by qRT-PCR, miRNA levels in whole urine of PCa patients were compared to patients with BPH and healthy controls. The investigators identified miR-1825 and miR-484 as potential urinary biomarkers for PCa diagnosis [135]. Unfortunately, this study was limited by a very small number of samples (8 PCa patients, 12 BPH patients, and 10 healthy men) [135]. In a profiling study, Sapre et al. identified miR-16, miR-21, and miR-222 as predictors of high risk PCa, but the authors were not able to validate the results in an independent cohort [136].

Due to their high stability and easy detection, urinary miRNAs have a high potential to become noninvasive biomarkers. Further investigations, using standard procedures for preanalytical processing and data normalization, followed by validation of candidate miRNAs in large clinical trials are required to translate incidental finding(s) into a clinically applicable test.

### 2.5. microRNAs as Predictive Biomarkers in Prostate Cancer

MiRNAs have also the potential to serve as predictive markers. In a profiling study, Selth et al. detected increasing levels of three circulating miRNAs (miR-141, miR-146b-3p, and miR-194) in serum of patients who experienced a rapid biochemical recurrence after radical prostatectomy, but only miR-146b-3p and miR-194 were also associated with disease progression in a validation cohort [119]. Santos et al. found higher levels of miR-221 and miR-7 in blood of patients diagnosed with high Gleason-score PCa and those patients with high miR-221 levels developed CRPC much earlier (10 versus 46 months) [137]. Furthermore, the authors observed a significantly lower overall survival in patients with high levels of miR-7 and, therefore, it is suggested that miR-221 and miR-7 can serve as potential predictive biomarkers in advanced prostate cancer [137]. Independently, Kneitz et al. reported that miR-221 in prostate cancer tissue is an independent predictor for cancer-related death and suggested that miR-221 offers a novel tissue-based predictive biomarker and possibly therapeutic target in high-risk PCa [138].

Recently, Huang et al. demonstrated that single-nucleotide polymorphisms (SNPs) in microRNAs or miRNA target sites can act as a useful predictive biomarker. The authors found two SNPs (rs2043556 in miR-605 and rs3737336 in the 3′UTR of* CDON*) to be associated with biochemical recurrence after radical prostatectomy (*P* < 0.05) [139]. SNP rs3737336 lays in* in silico* analysis within putative target sites of* CDON* for miR-181a, miR-181b, miR-181c, miR-181d, miR-4262, and miR-5007 [139]. Furthermore, the authors observed an increased risk for biochemical recurrence with cumulative number of risk alleles; two risk alleles and 3 or 4 risk alleles had 1.55-fold (*P* = 0.009) and 2.53-fold (*P* < 0.001) increased risk of biochemical recurrence, respectively [139]. Hulf et al. found that miR-205 DNA methylation is significantly associated with biochemical recurrence and the authors suggest that miR-205 is an epigenetically regulated tumor suppressor [83].

### 2.6. microRNAs as Therapeutic Response

The improvement of therapy modalities leads to a need for markers that can help decide the best therapy for the individual patient. In addition, markers are needed for monitoring, that is, to evaluate early therapeutic response, to monitor effectiveness of a treatment, and to predict chemoresistance [140, 141]. Only a few studies have investigated the levels of (circulating) miRNAs as therapeutic markers. The investigations that evaluated potential biomarkers in metastatic PCa and/or CRPC are limited by the number of patients and tested miRNAs [9, 109–111, 118]. In these studies, the researcher investigated upregulated circulating miRNAs in serum of CRPC patients that are applicable candidates as markers for therapeutic response. Nguyen et al. [109] demonstrated that miR-375, miR-378∗, and miR-141 were significantly higher in serum of CRPC patients compared to serum of low risk patients with a localized PCa. Zhang et al. [110] reported that serum levels of miR-21 are higher in patients with CRPC compared to patients with an androgen-dependent PCa and these patients with low levels of serum PSA had also levels of miR-21 similar to patients with a localized PCa or BPH [110]. Furthermore, Cheng et al. [111] confirmed the previous results and found that miR-141 (*P* < 0.0001), miR-200a (*P* = 0.007), miR-200c (*P* = 0.017), miR-375 (*P* = 0.009), and miR-210 (*P* = 0.022) were significantly elevated in serum of CRPC patients compared to age-matched controls [111].

Until now, only Lin et al. determined an association of circulating microRNAs and docetaxel chemotherapy outcome of CRPC patients (*n* = 97) [9]. The authors identified 14 miRNAs out of 46 to be associated with overall survival or PSA response to chemotherapy. Nonresponders to docetaxel and patients with a shorter survival had high levels of miR-200 family members prior to chemotherapy or decreased/unchanged levels of miR-17 family members after docetaxel treatment and therefore the authors associated high levels of miR-200 family members in serum with poor chemotherapy outcome of CRPC patients [9]. Furthermore, Gonzales et al. [118] analyzed miR-141, which is a member of the miR-200 family, together with circulating tumor cells (CTCs), lactate dehydrogenase (LDH), and PSA in serum of 21 PCa patients and examined the utility of miR-141 alone or in combination with CTCs, LDH, and PSA as a predictive marker and response to therapy. The authors found a strong correlation between clinical course according to progression and nonprogression of PCa and miR-141 levels. MiR-141 levels predicted a clinical progression with an odds ratio of 8.3 and had a high correlation with changes of PSA (*R* = 0.77; *P* < 0.001) and CTCs (*R* = 0.76; *P* < 0.001) [118]. However, independent studies are necessary to confirm these findings and to validate treatment response.

### 2.7. Extracellular Vesicles as Biomarkers in Prostate Cancer

It is known that cells are able to release several types of extracellular vesicles, which are involved in many physiological and pathological processes, such as immune response and cellular differentiation [142]. Extracellular vesicles differ mainly in their cellular origins and sizes. The most important extracellular vesicles released from cells are apoptotic bodies, exosomes, and shed microvesicles (MVs). Apoptotic bodies are released from the cell membrane as a final consequence of cell fragmentation during apoptosis [142]. They have an irregular shape and a size of 1–5 *μ*m [143, 144]. Exosomes have a size of 30–100 nm and are released by the fusion of multivesicular bodies (MVB) with the plasma membrane [143, 144]. Shed MVs, which are 100–1000 nm in size, are released by outward budding or blebbing of the plasma membrane [142]. Extracellular vesicles can be isolated from all body fluids, for example, blood, urine, semen, ascites, and malignant pleural effusion [125, 142] and they contain specific nucleic acids (e.g., miRNAs, mRNAs) and proteins, including enzymes, which represent their tissue origin.

Recent findings indicate that extracellular vesicles are not only waste products from cells. Exosomes and MVs that are released from viable cells are involved in intercellular communication in physiological as well as in pathological processes (e.g., cancer) [143]. Exosomes, actively secreted* in vitro* and* in vivo, *are involved in immune system modulation, regulation of neuronal cell functions, and cancer progression. Exosomes released from tumor cells can contribute to metastasis, stimulate angiogenesis, and can deliver prooncogenic miRNAs to target cells [142, 145–147]. All exosomes contain characteristic surface protein markers which enable their identification [6], such as CD9 [148], CD81 [149], and PDCD6IP [150].

Also MVs, which are characterized with high levels of phosphatidylserine in their membranes, contribute to cancer progression. MVs are, for example, able to modify the extracellular matrix through the involvement of lytic enzymes that are present in MVs [142, 151]. Through the transfer of enzymes MVs are also able to transform fibroblasts and epithelial cells to adopt typical cancer characteristics [152] and MVs can be involved in drug resistance through accumulation of antitumoral drugs [153].

In the literature another subpopulation of microvesicles, called prostasomes, has been described. Prostasomes are vesicles, derived from the prostate gland, that have a size of 50–500 nm (mean diameter 150 nm) and are present in high concentrations in seminal and prostatic fluids [154]. Some authors have hypothesized that prostasomes are just prostate-derived exosomes. Prostasomes are secreted by normal and malignant prostate acinar cells [155] after MVB fusion with membranes like exosomes, but they differ from exosomes according to their size and composition (e.g., membrane lipids). Like all extracellular vesicles, prostasomes are also involved in exchange of information. In physiological conditions, the recipient cells are mainly spermatozoa [156]. In addition, prostasomes are also involved in PCa progression [156] through an inhibition of the immune system, inhibition of the complement system, induction of migration by fibrinogen phosphorylation (protein kinases A and C), induction of invasion, and induction of angiogenesis [142]. Tavoosidana et al. evaluated the use of prostasomes as biomarkers and found elevated levels of these vesicles in semen of PCa patients, which were correlated with a higher Gleason-score of the tumor [157]. Furthermore, prostasomes were also found in plasma from PCa patients [142] suggesting their potential as biomarker.

Despite a clear difference in cell origin and size, extracellular vesicles are often overlapping according to their function and features. The currently available isolation techniques make it difficult to separate the subpopulations [142]. It was recently reported that tumor-derived circulating exosomes also contain miRNAs, which can downregulate their target genes in recipient cells [142]. Circulating exosomal miRNAs were correlated with miRNAs identified in tissue of ovarian cancer [158, 159] and lung cancer [160]. These results suggest that circulating exosomal miRNAs could potentially be used as diagnostic markers also in PCa [161]. The PCa specific gene fusion* TMPRSS2-ERG,* which is found in 50% of clinically localized PCa and which is associated with lethal PCa, is also present in exosomes isolated from VCaP cells (an androgen responsive PCa cell line) [162, 163]. Beside blood also urine, which contains exfoliated PCa cells and PCa-secreted products, is a promising resource for diagnostic markers. It is reported that urine from PCa patients contains more exosomes compared to patients with an indolent PCa or nonmalignant disease. In PCa patients, both* PCA3* and* TMPRSS2-ERG* were reported in urinary exosomes, showing that microvesicles may contain disease-specific molecules which then can serve as promising novel biomarkers [164]. However,* PCA3* and* TMPRSS2-ERG *were not detectable in exosomes from patients with ADT or in patients with bone metastasis after radical prostatectomy [164]. The currently used protocols for exosome isolation are time-consuming, including a series of (ultra) centrifugation steps and nanomembrane ultrafiltration. Further studies are necessary to establish new and faster methods for exosome isolation to enable fast and simple detection in routine clinical practice. The clinical relevance of circulating and urinary exosomes and prostasomes in semen as biomarkers for PCa needs to be evaluated.

### 2.8. Circulating Tumor Cells in Prostate Cancer

The importance of circulating tumor cells (CTCs) detection and molecular characterization is becoming evident. These cells can provide significant information for a better understanding of tumor biology and tumor cell dissemination [165]. CTC detection may be a useful approach for monitoring disease progression. CTCs have been shown to be the strongest independent predictor of overall survival in comparison to posttherapy PSA. CRPC patients with more than 5 CTCs in 7.5 mL blood have a significantly reduced overall survival compared to patients with less than 5 CTCs in 7.5 mL blood [166]. These results were confirmed in other studies [167, 168]. CTC detection remains a major technical challenge. Currently, the use for diagnosis and monitoring is limited by costly CTC isolation procedures and the low number of CTCs in blood. However, technical advances could lead to novel possibilities.

The clinical utility of monitoring CTCs is currently being tested in large phase III trials with PCa patients being treated with the novel antiandrogen abiraterone acetate or the novel androgen receptor antagonist enzalutamide (a.k.a. MDV3100). Molecular determinants can be identified and characterized in CTCs as potential predictive biomarkers of tumor sensitivity to a therapeutic modality [165, 169]. Potential miRNA biomarkers were also identified in CTCs. Sieuwerts et al. found that, for example, miR-183 is expressed in CTCs of metastatic breast cancer patients [170]. Whether CTCs of PCa patients express specific ncRNAs remains to be investigated.

## 3. Conclusion

To improve the management of PCa patients, novel diagnostic, predictive, and prognostic biomarkers are needed. The ideal biomarker should be able to detect the presence of a disease and predict its progression (i.e., identify high risk individuals), predict recurrence, and monitor therapy response. PCa and especially CRPC are heterogeneous diseases and, therefore, it is not expected that a single biomarker for all stages of the disease will be identified. Multiple biomarkers, or panels of markers, will be needed to address each particular clinical question.

The lncRNA* PCA3* has already been successfully translated into clinical setting, especially to predict (repeat) biopsy outcome of patients with elevated serum PSA. Recent studies showed the potential of other ncRNAs, especially the miRNAs, as novel biomarkers for PCa. Across independent studies, elevated serum levels of miR-141 and miR-375 have been consistently found and confirmed for metastatic PCa and/or CRPC. These miRNAs are associated with further prognostic parameters like higher Gleason-score and positive lymph node status. There are a number of additional promising miRNAs and other biomarkers, such as exosomes and CTCs, for PCa on the horizon, but these await further validation in independent studies. Only then, these biomarkers can be translated into clinical practice.

## Figures and Tables

**Table 1:** Overview of prostate cancer biomarkers.

Specimens	Category	Examples	Up/down	Summary/description	Reference
Tissue	**Noncoding RNAs:**				
	lncRNAs	*PCA3 *	*↑ *	PCa-specific, 66-fold upregulated in PCa-tissue compared to nonmalignant prostate tissue (in >95% of PCa patients)	[6, 18, 40–43]
		*CBR3-AS1, MALAT1, PCAT1, 6, 7, 18, PCGEM1, PRNCR1,* and* SCHLAP1 *	*↑ *	Identified overexpressed lncRNAs in PCa tissue	[23, 44–51]
	microRNAs	miR-21, miR-183/96/182	*↑ *	Well-known oncomirs, upregulated miRNAs in several cancer tissues	[52–71, 108]
		miR-221/222	*↑ *	Upregulated in different cancer tissue, highly expressed in CRPC tissue	[66, 72, 73]
		miR-375	*↑ *	Upregulated in PCa tissue compared to nonmalignant tissue	[66, 109]
		miR-143/145	↓	Downregulation is associated with progression of cancers	[91–93]
		miR-205, miR-200-family	↓	Well-known tumor suppressor, downregulated in many cancer tissues, involved in EMT	[66, 74–90]
	**Protein-coding genes:**				
	mRNAs/proteins	AMACR, caveolin-1, CD147, endoglin (CD105), human kallikrein-2, interleukin-6, PSMA, and TGF-*β* 1		Upregulated in PCa tissue compared to nonmalignant tissue; AMACR: upregulated in 88% of PCa, CRPC and metastasis strongly positive; CD147: overexpressed in many solid tumors; increased expression is associated with PCa progression and poor prognosis; PSMA: transmembrane glycoprotein, upregulated in PCa tissue compared to benign tissue; TGF-*β* 1: growth factor; increased expression correlates with tumor invasion, metastasis and biochemical recurrence; human kallikrein-2: serine protease activates pro-KLK3 to its active form (PSA)	[6, 18]
	**DNA modifications:**				
	Genetic modification	*TMPRSS2-ERG* fusion, BRCA1/2 mutation		*TMPRSS2-ERG* gene fusion: expressed in malignant prostate tissue, independent marker of disease progression and poor prognosis; BRCA1/2 are tumor suppressors; BRCA2 mutation is associated with aggressive PCa and poor survival outcome	[6, 18]
	Epigenetic modification	*GSTP1, RASSF1A *		*GSTP1* hypermethylation was detected in 90% of prostate cancer; *RASSF1A* is a tumor suppressor gene; *RASSF1A* hypermethylation has been observed in 60–74% of PCa and in 18.5% of BPH	[18, 19]

Body fluids (blood and/or urine)	**Noncoding RNAs:**				
	lncRNAs	*PCA3 *	*↑ *	PCA3 score (*PCA3/KLK3* ratio) FDA approved as diagnostic biomarker for PCa (sensitivity 66%, specificity 76%)	[6, 18, 41–43]
		miR-141, miR-375, miR-21, miR-221/222	*↑ *	upregulated in plasma/serum of PCa patients with advanced disease (metastasis and/or CRPC)	[108–130]
	microRNAs (circulating)	miR-200-family	*↑ *	Upregulated in serum of CRPC patients; high levels were found in serum of nonresponders to docetaxel prior treatment, associated with shorter survival	[9, 111]
		miR-107, miR-574-3p	*↑ *	Upregulated in urine of PCa patients compared to healthy controls	[112]
		miR-205, miR-214	↓	Downregulated in urine of PCa patients compared to healthy controls	[134]
	**DNA modifications:**				
	Genetic modification	*TMPRSS2:ERG* fusion		Detection of TMPRSS2:ERG fusion transcript in urinary sediments, obtained after DRE (specificity 93%), combined test: PCA3 score + *TMPRESS2:ERG *fusion in urine after DRE—improved sensitivity from 66% (PCA3 alone) to 73% (combined)	[6, 18]
	Epigenetic modification	*GSTP1 *		*GSTP1 *hypermethylation was found in postprostate massage urine sediments of 68% of PCa patients with early confined disease, 78% of patients with locally advanced PCa, 29% of patients with PIN, and 2% of patients with BPH.	[18, 19]
	**Cells/vesicles:**				
	CTCs			Detection of CTCs in blood has the potential to evaluate disease progression and for monitoring of therapy response. The Veridex CellSearch Assay has received FDA approval for the enumeration of CTCs in prostate cancer.	[16, 165–170]
	Extracellular vesicles	Exosomes, apoptotic bodies, microvesicles, and prostasomes		Extracellular vesicles are cell-derived vesicles that can be isolated from urine and blood and have the potential as biomarker for PCa. They also contain specific DNA, RNA, and protein molecules that are unique to the cells they originate of, and these could also serve as biomarker(s).	[125, 142–150, 157]

AMACR: *α*-methylacyl-CoA racemase; CTCs: circulating tumor cells; *GSTP1*: glutathione S-transferase pi 1; KLK3: kallikrein-3; lncRNAs: long noncoding RNAs; PCa: prostate cancer; PSA: prostate-specific antigen; PSMA: prostate-specific membrane antigen.
